# Process Optimization and Quality Improvement of Fermented Foods and Beverages

**DOI:** 10.3390/foods14071238

**Published:** 2025-04-01

**Authors:** Tiziana Di Renzo, Anna Reale

**Affiliations:** Institute of Food Sciences, National Research Council (CNR-ISA), Via Roma 64, 83100 Avellino, Italy; anna.reale@isa.cnr.it

## 1. Introduction

The optimization of production processes and the evolution of microbial fermentation techniques have significantly contributed to the advancement of modern food processing. Microbial fermentation has been used since ancient times for food preservation and to improve sensory properties. Over time, fermentation, which was historically an empirical process relying on the activity of spontaneous microbes and uncontrolled environmental conditions, has evolved with the use of starter cultures and controlled environments, improving the quality and safety of fermented foods. Overall, advancements in microbial fermentation are not only aimed at better preserving food, thereby improving sensory and nutritional characteristics, but also at contributing to sustainable practices by reducing waste and energy consumption.

Microorganisms such as bacteria, yeasts, and moulds play a crucial role in fermentation processes, carrying out complex biochemical reactions that significantly impact the overall quality of food products [1,2,3]. The metabolites produced during fermentation (such as lactic and acetic acid, alcohol, and other minor compounds) can contribute to improve food safety by inhibiting the growth of spoilage microorganisms and contributing to a richer flavour profile. Furthermore, fermentation can lead to the production of bioactive compounds such as peptides, vitamins, and antioxidants, which can have positive effects on human health [4]. Consequently, scientific and industrial interest is increasingly being directed towards microbial-driven fermentations for the formulation and optimization of new fermented foods and beverages. However, despite the favourable properties of fermentation and traditional microorganisms used in food production, several challenges remain that require process optimization. The main technological advancements are aimed at optimizing conditions to increase the yield of desired compounds or products during fermentation while reducing waste, energy consumption and environmental impact.

## 2. An Overview of Published Articles

Based on these assumptions, this Special Issue collected fourteen contributions (thirteen articles and one review) on different food matrices (cocoa, pistachio, rice, barley, quinoa, vinegar, fish and kombucha), highlighting the importance of optimizing fermentation processes to improve quality characteristics.

Five contributions (numbered 1, 2, 3, 4, and 5) focused on enhancing the quality characteristics of cocoa, which plays a key role in the world economy due to its widespread use in the production of many different foods, such as biscuits, chocolate spreads, beverages and confectionery [5]. Streule et al. (contributions 1 and 2) provide insights on post-harvest techniques and fermentation control, highlighting the importance of careful management throughout the cocoa supply chain in order to produce high-quality products. In particular, the authors emphasize the importance of post-harvest techniques (pod storage and drying temperatures) and the control of fermentation parameters in determining the quality and sensory properties of Ecuadorian cacao. Controlling drying temperatures helps to mitigate undesirable sensory characteristics, such as excessive bitterness and astringency, in the final products, ensuring consistent quality, even in different geographical locations. In addition, the authors emphasized the importance of monitoring the conditions involved in cocoa fermentation, such as temperature, microbial activity, fermentation duration, pH levels and drying techniques, to avoid off-flavours and improve the texture and flavour profiles of cocoa products, ultimately meeting international market standards for cocoa. In this regard, Guzmán-Armenteros et al. highlighted that the use of electromagnetic fields (EMFs) in cocoa fermentation is an emerging research area with promising potential for improving product quality (contribution 3). Their study evaluated the potential effects of low-frequency electromagnetic fields (EMFs) on the fermentation process of cocoa beans, with the aim of improving cocoa production, which is a crucial step in the production of chocolate (contribution 4). EMFs have been shown to positively influence microbial activity during fermentation, increasing enzyme production and improving the overall quality of cocoa beans. However, although the study shows a promising effect, the exact biological mechanisms behind how EMFs interact with microbial ecosystems remain unclear. Therefore, more detailed investigations into the underlying biological interactions between microorganisms and electromagnetic fields are required to establish the optimal conditions for the application of EMFs in cocoa fermentation processes.

In contribution 5, the authors evaluated the effect of post-harvest processes on the physico-chemical composition and sensory properties of cocoa beans, with a focus on techniques such as solar pre-drying and controlled fermentation using a yeast starter. The results showed that outdoor solar pre-drying of cocoa beans can reduce acidity and improve flavour. In addition, the use of selected yeast cultures during fermentation could help manage acidification more effectively, leading to a higher-quality cocoa with more desirable sensory properties. It is clear from the contributions collected that the quality of cocoa beans is influenced by a series of carefully managed processes, from agricultural practices to industrial processing stages. Each step, such as agricultural practices, post-harvest techniques and fermentation conditions, plays a crucial role in determining the final taste profile and quality of chocolate products. By optimizing these steps through innovative techniques such as EMFs or advanced drying technologies, producers can enhance both product consistency and sensory attributes of chocolate products.

This Special Issue also collects three studies on the development of new fermented beverages, highlighting innovative approaches in fermentation technology. In particular, in contribution 6, the authors explored the use of *Bacillus amyloliquefaciens* as an alternative to the traditional *Aspergillus oryzae* for producing amazake, a traditional sweet Japanese rice-based beverage. During fermentation, the starch is broken down into sugars, giving the beverage its characteristic sweetness without added sugars. Two additional studies investigated the use of lactic acid bacteria (LAB) for the production of kombucha and pistachio beverages. Anderson et al. (contribution 7) explored the use of LAB cultures combined with backslopping technology to ferment kombucha in alternative matrices such as orange juice and coffee infusion. Introducing new substrates altered the microbial community, changing both chemical and sensory profiles of the resulting beverages. The new kombucha samples showed improved sensory characteristics, suggesting a distinct appeal compared to traditional ones. The backslopping played a key role in modifying the microbial consortia, potentially speeding up the fermentation process while maintaining flavour consistency and quality.

Reale et al. (contribution 8) on the other hand, confirmed the suitability of LAB in fermenting a pistachio-based beverage, as already pointed out by other authors [6]. The use of LAB led to unique flavours with pronounced acidity and volatile compounds such as acetoin and 2,3-butanedione, contributing to a yoghurt- or kefir-like aroma. Furthermore, the use of a colloidal mill to grind and homogenize the pistachios proved to be an innovative way to improve beverage consistency and reduce waste, representing an example of how food processing technology can contribute to improve both product quality and sustainability. The fermented pistachio beverages obtained offers a rich combination of protein, healthy fats, fibre and key minerals such as potassium and phosphorus. Furthermore, as also highlighted by Garzon et al. [7], the fermentation of pistachio beverages with LAB increased the bioaccessibility of minerals, improving the nutritional quality of the pistachio-based beverage. This type of beverage can be considered a nutritious option, especially for those seeking plant-based protein sources or who wish to increase their intake of essential minerals.

The study by Kim et al. (contribution 9) on the use of LAB to ferment barley sprouts highlights the potential anti-inflammatory effects of fermented foods, particularly in relation to gut health and inflammation management, offering promising new insights into the role of selected LAB species and bioactive compounds in managing diseases related to intestinal inflammation.

Contribution 10 presents an interesting and efficient breakthrough to improve the production process of Zhenjiang aromatic vinegar, a staple food in Chinese cuisine. The study introduced a dry gelatinization process to solve inefficiencies in the traditional production of Zhenjiang aromatic vinegar, such as high water consumption, waste water generation, raw material waste and limited mechanization. The results showed an improvement in alcohol production efficiency and vinegar yield, as well as an improvement in umami flavour and increased levels of non-volatile organic acids. Furthermore, the dry gelatinization process is distinguished by its brief duration of gelatinization and high level of automation, presenting a decisive advantage in operational and economic efficiency as well as process control.

The study by Di Renzo et al. (contribution 11) highlights the potential of quinoa flour as a base for gluten-free breads, emphasizing the importance of using hydrocolloids to optimize texture and gas retention during fermentation. The authors highlighted that quinoa flour, with its nutritional profile, can be an excellent base for enhancing the nutritional profile of gluten-free breads. Furthermore, they tested different hydrocolloids, such as HPMC (hydroxypropyl methylcellulose), xanthan gum and others, highlighting that each hydrocolloid has specific advantages at different concentrations, leading to different results in terms of bread volume, crumb consistency and even flavour. Overall, the authors underscore the potential benefits of combining quinoa flour with appropriate hydrocolloids to create high-quality gluten-free breads that meet both nutritional needs and consumer preferences.

In addition, this Editorial includes two contributions (12 and 13) on the optimization of high-density fermentation of *Saccharomyces fibuligera* Y1402 and the traditional solid-state fermentation of Chinese liquor using statistical models. In the first study, Response Surface Methodology (RSM) was used to optimize culture medium and fermentation conditions for *Saccharomyces fibuligera* Y1402, addressing a critical gap in its industrial application. These optimizations significantly enhance the production efficiency and yield of valuable metabolites, enzymes, or other bioproducts from *S. fibuligera* Y1402, supporting more efficient and cost-effective biotechnological processes (contribution 12). In contrast, the study by Jin et al. (contribution 13) delves into the complexities of solid-state fermentation (SSF), in particular its use in Chinese liquor production. By using statistical models to analyze industrial data and uncover hidden factors such as the influence of the starting month and the role of lactic acid bacteria, the authors have provided both theoretical insights and practical guidance that can help improve fermentation practices. The study not only aids in the optimization of the SSF process but also paves the way for more advanced research, combining microbiology and engineering to ensure that traditional fermentation processes can be scientifically validated and improved.

Finally, contribution 14 shows that sea urchins can be successfully used in the production of fish sauces. Both whole and shelled sea urchins contribute essential microbial and enzymatic activity during fermentation, leading to the hydrolysis of fish proteins and the development of a high-quality product. Sensory properties, in particular, the unique aromas of crustaceans and molluscs, also make sea urchin-enhanced sauces stand out in terms of flavour, opening up the possibility of innovative culinary products. This study paves the way for further exploration of sea urchin ingredients in fermented foods and sauces.

## 3. Conclusions

The growing demand for functional foods has led to increased scientific and industrial interest in microbial fermentation processes. Research increasingly focuses on the development of new fermentation processes that can produce safe, healthy and functional foods with specific health-promoting properties. This Special Issue analyzed different approaches and innovations in the areas of process optimization and quality improvement in fermented foods and beverages. The research highlights not only the potential for improved health benefits, such as the selection of LAB species for the treatment of diseases related to intestinal inflammation, but also the ability of certain technological interventions to reduce waste and energy consumption. The analysis of the collected articles shows that optimizing fermentation processes and improving food quality both require a comprehensive approach that includes several key factors, such as the selection of raw materials and microbial strains, the control of fermentation parameters, and technological innovations. By improving these factors, product consistency, nutritional value, safety and overall consumer satisfaction can be improved. Moreover, with the increasing focus on sustainability, these innovations could be key to making the food industry more environmentally friendly.

## References

[1] Graham A.E., Ledesma-Amaro R. (2023). The microbial food revolution. Nat. Commun..

[2] Kumar V., Ahire J.J., Taneja N.K. (2024). Advancing microbial food safety and hazard analysis through predictive mathematical modelling. Microbe.

[3] Sawant S.S., Park H.-Y., Sim E.-Y., Kim H.-S., Choi H.-S. (2025). Microbial Fermentation in Food: Impact on Functional Properties and Nutritional Enhancement—A Review of Recent Developments. Fermentation.

[4] Anumudu C.K., Miri T., Onyeaka H. (2024). Multifunctional applications of lactic acid bacteria: Enhancing safety, quality, and nutritional value in foods and fermented beverages. Foods.

[5] Moussoyi Moundanga S., Petit J., Ndangui C.B., Scher J., Nzikou J.-M. (2024). Impact of cocoa variety on merchant quality and physicochemical characteristics of raw cocoa beans and roasted cocoa mass. Discover Food.

[6] Di Renzo T., Osimani A., Marulo S., Cardinali F., Mamone G., Puppo M.C., Garzon A.G., Drago S.R., Laurino C., Reale A. (2023). Insight into the role of lactic acid bacteria in the development of a novel fermented pistachio (*Pistacia vera* L.) beverage. Food Biosci..

[7] Garzòn A.G., Puppo M.C., Di Renzo T., Drago S.R., Reale A. (2025). Mineral bioaccessibility of pistachio-based beverages: The effect of lactic acid bacteria fermentation. Plant Foods Hum. Nutr..

